# Evaluating [^18^F]-DCFPyL for Detecting Prostate Cancer Recurrence: A Cost–Consequence Comparison with Alternative PET Radiotracers in Spain

**DOI:** 10.3390/jmahp14010007

**Published:** 2026-01-23

**Authors:** Tiago Matos, Mrunmayee Godbole, Rithvik Badinedi, Madhusubramanian Muthukumar, Marina Hodolic, Nicolas Tchouen, Anthony Berthon

**Affiliations:** 1 Alira Health, Global Health Economics, 4057 Basel, Switzerland; tiagofvmatos@gmail.com; 2 Alira Health, Global Health Economics, 08029 Barcelona, Spain; 3 Alira Health, Global Health Economics, London WC1V 6DF, UK; rithvik.badinedi@alirahealth.com (R.B.); madhusubramanian.muthukumar@alirahealth.com (M.M.); 4 Curium, 75008 Paris, France; marina.hodolic@curiumpharma.com (M.H.); nicolas.tchouen@curiumpharma.com (N.T.); anthony.berthon@curiumpharma.com (A.B.)

**Keywords:** [^18^F]-DCFPyL, prostate cancer, biochemical recurrence, BCR, PET/CT, cost–consequence

## Abstract

**Introduction:** [^18^F]-DCFPyL (Piflufolastat [^18^F]) is a prostate-specific membrane antigen (PSMA)-targeted position emission tomography (PET) radiotracer for detecting the biochemical recurrence (BCR) of prostate cancer (PCa). This study evaluates its economic impact compared with [^68^Ga]-PSMA-11, [^18^F]-FCH, and [^18^F]-PSMA-1007 from the Spanish National Healthcare System’s perspective. **Methods:** A cost–consequence model, over a 5-year time horizon, simulated the diagnostic and treatment pathway based on radiotracer-specific accuracy and disease localization. Treatment options included a radical prostatectomy, radiation therapy, androgen deprivation therapy (ADT), and radiation therapy + ADT. Costs were calculated for true/false positives and negatives. Due to limited data availability, key inputs were informed by expert opinions, supported by published meta-analyses, public sources, and literature. Officially published Spanish prices were applied: EUR 2000 for [^18^F]-DCFPyL, [^68^Ga]-PSMA-11, and [^18^F]-PSMA-1007, and EUR 1144 for [^18^F]-FCH. **Results:** The use of [^18^F]-DCFPyL led to fewer unnecessary therapies; specifically, it led to 11,229 (74%) fewer than [^68^Ga]-PSMA-11, and 5180 (56%) and 7771 (66%) fewer than [^18^F]-FCH and [^18^F]-PSMA-1007, respectively. It achieved significant cost savings for repeated testing: EUR 15M (43%) versus [^68^Ga]-PSMA-11, EUR 37M (65%) versus [^18^F]-FCH, and EUR 27M (58%) versus [^18^F]-PSMA-1007. Cost savings for false positives were EUR 15M (50%) against [^68^Ga]-PSMA-11, EUR 22M (60%) versus [^18^F]-FCH, and EUR 29M (66%) compared with [^18^F]-PSMA-1007. The cost per correct diagnosis was reduced by EUR 198 (8%) compared with [^68^Ga]-PSMA-11 and EUR 377 (15%) relative to [^18^F]-PSMA-1007, while showing a EUR 635 (40%) increase compared with [^18^F]-FCH. **Conclusions:** [^18^F]-DCFPyL offers a cost-saving option for BCR detection within the Spanish National Healthcare System by reducing the number of unnecessary therapies, the cost of false positives, and repeat testing compared with alternative radiotracers. These improvements support the potential for better diagnostic outcomes and more informed downstream clinical decision-making.

## 1. Introduction

Prostate cancer (PCa) is the second most diagnosed malignancy among males worldwide, with approximately 1.4 million new cases in 2020, contributing to approximately 375,000 deaths, making it the fifth leading cause of cancer-related mortality in men [1]. In Spain, PCa remains the most prevalent cancer among men, accounting for an estimated 29,002 new cases in 2023 (18% of all male cancer diagnoses), with an overall patient prevalence of approximately 259,788 being reported in 2020 [2]. A retrospective Spanish study conducted by Darba et al. in 2024 reported a median age of 72 years among PCa patients. The majority (66%) of cases were observed in individuals aged 66 to 85 years old; 25% were aged 45 to 65 years old, and 9% were aged over 85 years old [3]. Despite receiving early treatment, between 20% and 50% of men with PCa experience biochemical recurrence (BCR) within 10 years of the initial definitive therapy, marked by increasing serum prostate-specific antigen (PSA) levels [4] without a detectable metastatic disease on conventional imaging, thus complicating clinical decision-making [5,6].

Recent advancements in prostate-specific membrane antigen (PSMA)-targeted radiotracers have significantly enhanced the detection of BCR. These radiotracers, which selectively bind to PSMA, a glycoprotein expressed 100 to 1000 times more on PCa cells compared with normal prostate tissue [7], enable whole-body imaging of cancer spread using positron emission tomography (PET) [8]. This improved sensitivity and specificity [9,10] represents a substantial step forward with the introduction of advanced radiotracers, such as [^18^F]-DCFPyL, [^68^Ga]-PSMA, and [^18^F]-PSMA-1007, exhibiting varying levels of diagnostic accuracy, sensitivity, and specificity. However, PSMA expression demonstrates significant inter- and intra-patient heterogeneity. A study by Yaxley et al. [11] found that 5% to 10% of primary tumors are PSMA-negative based on immunohistochemistry (IHC) results, and approximately 10% are negative on PSMA-PET despite elevated PSA levels. Based on the study by Ferraro et al. [12], the presence of PSMA-negative areas within the primary tumor correlates with negative PSMA-PET scans for BCR. Integrating quantitative PSMA expression data from molecular pathology of the primary tumor with clinical factors enhances patient selection and reduces the occurrence of false-negative PET scans. Before the introduction of PSMA radiotracers, PET with [^18^F]-FCH was increasingly utilized to differentiate between locoregional and distant metastases in patients with BCR, particularly in those at intermediate to high risk [13]. With PSMA PET/computed tomography (CT) becoming increasingly integrated into clinical practice, it is essential to determine whether the diagnostic benefits justify the associated costs in healthcare settings [8]. Currently, comparative data on the clinical and economic performance of available PSMA radiotracers are lacking, making it challenging to identify the optimal radiotracer for precise disease detection [14] and to inform the subsequent line of treatment [15].

Among the promising PSMA-targeted agents, [^18^F]-DCFPyL has gained attention due to its distinct imaging characteristics and clinical impact, receiving approval from the Food and Drug Administration (FDA) in May 2021 [16] and from the European Medicines Agency (EMA) in November 2023 [17] for use in adult male patients with PCa. It is indicated for the detection of PSMA-positive lesions via PET in adult male patients with PCa, specifically for primary staging of those with high-risk disease prior to initial curative therapy, as well as for the localization of recurrent disease in patients with suspected recurrence based on rising PSA levels following curative-intent treatment. The efficacy and safety of [^18^F]-DCFPyL were evaluated in three prospective, open-label, multicenter studies involving men with PCa: OSPREY (NCT02981368) [18], CONDOR (NCT03739684) [19], and PYTHON (EudraCT number 2020-000121-37) [20,21].

Across the OSPREY, CONDOR, and PYTHON studies, [^18^F]-DCFPyL consistently demonstrated relatively better diagnostic performance in detecting PCa BCR and metastases. In the OSPREY study [18] for pelvic lymph node metastasis detection, sensitivity ranged from 31% to 42%, and specificity from 96% to 99%. In a separate cohort, sensitivity ranged from 93% to 99%, and regional-level analyses showed sensitivity ranges of 93% to 100% for pelvic lesions and 91% to 98% for extra-pelvic lesions. The CONDOR study, [19] involving 208 men with a median PSA of 0.8 ng/mL, reported a high composite likelihood ratio of 85% to 87%, along with a disease detection rate of 59% to 66% and a change in intended management in 64% of patients. In the PYTHON study [20,21], [^18^F]-DCFPyL showed a significantly higher detection rate (58%) compared with [^18^F]-FCH PET/CT (40%; *p* < 0.0001), with detection increasing with higher PSA values and a greater impact on patient management being noted (44% vs. 29%). Across the studies, no drug-related or serious adverse events were reported, reinforcing the safety and diagnostic value of [^18^F]-DCFPyL in PCa management.

A previous cost–consequence analysis by Jensen et al. [22] aimed to quantify the economic impact and cost consequence of using ^18^F-fluciclovine PET/CT in PCa BCR, compared with conventional imaging modalities, including magnetic resonance imaging (MRI), CT, and single-photon emission computed tomography (SPECT). The current study is the first comprehensive study aiming to evaluate the economic impact, via a cost–consequence analysis, of using [^18^F]-DCFPyL in comparison with other radiotracers for the detection and localization of recurrent PCa, from the perspective of the Spanish National Healthcare System. In addition, while [^18^F]-DCFPyL is indicated for primary staging, this analysis focuses solely on the detection and localization of BCR due to limited data availability for primary staging. The results of this study have the potential to inform both clinical guidelines and resource allocation decisions in PCa management in Spain.

## 2. Methods

### 2.1. Overall Approach

A cost–consequence analysis (CCA) was conducted, based on the diagnostic and treatment pathways outlined in the current European Society of Medical Oncology (ESMO) Clinical Practice Guidelines for PCa [23]. A CCA was selected for two main reasons. First, no head-to-head randomized controlled trials comparing [^18^F]-DCFPyL with other PSMA-targeted radiotracers are available, and the evidence base for each tracer varies substantially in study design, patient characteristics, and reported outcomes. These limitations precluded the development of a full cost-effectiveness analysis (CEA), linking diagnostic performance to downstream health outcomes, such as life years or quality-adjusted life years (QALYs). Second, given these evidence constraints, a CCA allows for transparent presentation of disaggregated outcomes, including total costs related to false positives, costs of repeat testing, number of correct diagnoses, and number of unnecessary therapies, without requiring assumptions regarding long-term disease progression that could not be robustly supported by the literature. This approach provides decision-makers with a clear understanding of the immediate economic and diagnostic implications of adopting [^18^F]-DCFPyL relative to alternative tracers ([^68^Ga]-PSMA-11, [^18^F]-FCH, and [^18^F]-PSMA-1007), which were selected for detecting BCR through PET/CT based on their clinical relevance and availability in practice. To further inform setting-specific inputs and validate model assumptions, semi-structured interviews were conducted with two Spanish clinical experts specializing in PCa and confirmed with one health economic expert. These interviews provided insights into local diagnostic pathways, epidemiology, and resource use within the Spanish National Healthcare System.

### 2.2. Semi-Structured Expert Interviews

Two medical experts from Spain, comprising a nuclear medicine physician and a urologist, were considered sufficient to provide primary model inputs in areas where published evidence was limited or unavailable. They were selected for their extensive clinical experience, leading roles in high-volume centers, and direct involvement in managing the target patient population, ensuring that their insights reflected current and representative clinical practice. While a larger expert panel could have broadened the range of perspectives, the parameters informed by expert opinion related primarily to operational or practice–pattern assumptions that typically show limited variation across centers. Moreover, all expert-derived inputs were tested in sensitivity analyses to account for any potential variability and uncertainty.

Experts were invited via email to participate in 90 min interviews, were provided with study details and a consent form, and were requested to submit their insights. Interviews were recorded for transcription, and participants were compensated according to fair market value in Spain. The aim of the interviews was to gather primary data on the patient and treatment pathways in Spain; the treatment mix for patients suffering from local, regional, or metastatic PCa; the epidemiology of PCa in Spain; and diagnostic and treatment costs. Model inputs, model structure, and key model assumptions were validated with the experts, and conflicting opinions were addressed by undertaking a one-way sensitivity analysis.

### 2.3. Patient Population

The simulated population for this analysis comprised men aged 60 to 80 years old with histologically confirmed PCa presenting with BCR, defined as a rise in levels of PSA in the blood after a radical prostatectomy and radiotherapy. Disease stages in the model were categorized into localized high-risk, regional spread, metastatic hormone-sensitive prostate cancer (mHSPC), and metastatic castration-resistant prostate cancer (mCRPC). The likelihood of BCR was estimated to be 20% to 40% based on published data [24].

### 2.4. Model Structure

**Figure 1:**
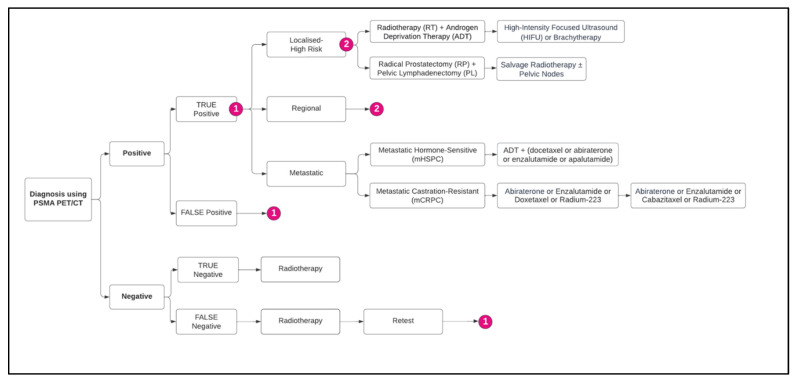
Model structure of BCR diagnoses and treatment pathways. Note: 1: Classification of PCa into localized high-risk, regional, and metastatic. 2: Treatment of PCa either with RT + ADT or RP + pelvic lymphadenectomy. Abbreviations: PSMA PET/CT: prostate-specific membrane antigen positron emission tomography/computed tomography; RT: radiotherapy; ADT: androgen deprivation therapy; HIFU: high-intensity focused ultrasound; RP: radical prostatectomy; PL: pelvic lymphadenectomy; mHSPC: metastatic hormone-sensitive prostate cancer; mCRPC: metastatic castration-resistant prostate cancer.

A decision tree model (Figure 1) was constructed in Microsoft Excel (version 2402, Microsoft Corporation, Redmond, WA, USA) to represent the diagnostic and treatment pathways for PCa using PET/CT. A decision analytic tree was chosen over other methodologies, as the value of [^18^F]-DCFPyL lies in the first year of treatment, and the transition probabilities required to populate the Markov model were not available due to the lack of a head-to-head randomized controlled trial; therefore, the model would have largely been based on assumptions.

This analysis considered 1 year of patients with PCa recurrence and incidence presenting with suspected local, regional, or advanced/metastatic disease. The rationale for limiting the follow-up period to the first year was based on the understanding that the benefits of PSMA radiotracers in diagnosing BCR are most pronounced during initial patient management, potentially altering treatment strategies within the first 6 months following the diagnosis, and the treatment outcomes thereafter.

The analysis was conducted from the perspective of the Spanish National Healthcare System. In the base case, the model assumed a period of 5 years to account for the cost consequence of utilizing [^18^F]-DCFPyL for the detection of BCR. However, the model provided the user with the flexibility to vary the time horizon from 1 to 10 years, because the likelihood of BCR within a decade ranges between 27% and 53%. Approximately 30% to 50% of patients who undergo radiotherapy (RT) or 20% to 40% of patients initially treated with a radical prostatectomy (RP) may subsequently experience BCR within 10 years [25]. Patients were classified based on diagnostic PET/CT test results as either positive or negative, which further determined their subsequent treatment pathways. Those with positive test results were subdivided into true positives or false positives and progressed through the stages of localized high-risk, regional, or metastatic disease according to diagnostic accuracy, while those with negative test results were subdivided into true negatives or false negatives. Figure 1 illustrates the decision tree pathways, highlighting the diagnostic and treatment scenarios for different patient classifications.

### 2.5. Treatment Pathways

The treatments for localized and regional PCa included RP with a pelvic lymphadenectomy (PL), RT, ADT, RT + ADT, external beam radiotherapy (EBRT), brachytherapy, or a combination of the treatments. The model accounted for the treatment costs incurred due to true positives, false positives, true negatives, and false negatives, based on the test specifications of the radiotracers.

Patients with a confirmed true positive test result were assumed to receive stage-specific treatment, including combinations of RT + ADT or RP + PL. A simplifying assumption, based on expert opinion, was that for those initially identified as false positives, a partial course of treatment was modeled for a period of 6 months until retesting confirmed the absence of disease, leading to therapy discontinuation. Based on the expert clinical opinion, patients who had a false negative initially received salvage radiation therapy for a period of 6 months as a prophylactic measure, followed by retesting to determine the appropriate management strategy. Patients with mHSPC received a combination of either ADT and novel hormone agents (NHAs, including abiraterone, enzalutamide, or apalutamide) or chemotherapy (docetaxel) and an NHA [26]. Patients with mCRPC received either one of the NHAs (abiraterone, enzalutamide) or a combination of an NHA and chemotherapy (docetaxel, cabazitaxel) or ^223^Ra (Radium-223) for patients unfit for the above treatments (and bone-only metastases) [27]. For simplicity, it was assumed that each false positive and false negative patient underwent a single IHC test to confirm either a true positive or true negative outcome. Additionally, patients with false-positive results were assumed to undergo a 6-month treatment period before a definitive negative diagnosis was confirmed. These assumptions were based on expert clinical input from urologists and nuclear medicine specialists, ensuring that the model remained representative of real-world practice while limiting complexity. All expert inputs were adjusted by ±20% to establish upper and lower bounds, capturing the uncertainty associated with the point estimates provided by the experts, and their impact on the outcomes was tested via a one-way sensitivity analysis (Supplementary Materials).

### 2.6. Diagnostic Test Performance Specifications

The CCA leverages the sensitivity and specificity of each radiotracer to estimate the number of true positives, true negatives, false positives, and false negatives to predict the health outcomes of the testing. Based on the test specifications for different comparators, the analysis considered the number of unnecessary treatments potentially avoided and the associated cost implications of undertaking PET/CT using [^18^F]-DCFPyL versus comparators. A literature review was conducted to identify the diagnostic performance, including the sensitivity and specificity (Table 1), safety, and economic burden of [^18^F]-DCFPyL and its comparators.

### 2.7. Outcomes Considered

The primary outcomes of the CCA were cost per correct diagnosis; the number of unnecessary treatments, defined as the unnecessary treatments resulting from diagnostic errors, such as false positives or the therapies that were omitted or inappropriately planned based on false-negative imaging results [22], incurring an additional cost of repeated testing; and the cost of treating false positives and false negatives. The secondary outcome was the total correct diagnosis, defined as the total of all true positive and true negative imaging test results.

### 2.8. Economic Inputs

The acquisition costs for all the tracers were sourced from the Spanish public maximum ceiling price published by the Commission on Medicine Prices (Comisión Interministerial de Precios de los Medicamentos, [CIPM] 177, 238, 246, 249) [32]. The treatment costs [32,33,34,35,36,37,38,39,40,41,42] are listed in the Supplementary Materials. Please note that the Value-Added Tax (VAT) and indirect costs were not included. The cost of re-testing was applied to patients who had tested positive (but were false positives) and those who tested negative (but were false negatives). Unnecessary treatments pertained to those who, having tested positive, were later confirmed to be false positives and were treated for a short amount of time (6-month assumption in the model) until they stopped the treatment. The model assumed that the costs of hormone and radiation therapy combination matched the cost of RT, plus 50% of the hormone therapy costs [22].

### 2.9. Sensitivity Analysis

A deterministic one-way sensitivity analysis (DSA) was conducted to assess the robustness of the model’s outcomes. All model inputs were varied by ±20% to examine the impact of parameter uncertainty on the results. This range was selected to capture plausible variations in the model inputs, which were primarily informed by expert opinion. Additionally, a scenario analysis was conducted to enhance the robustness of the results. Two scenarios were considered: The first involved extending the time horizon from 5 years, as used in the base case, to 10 years. The second involved adjusting the probability of BCR from 30% in the base case to 50% in the scenario, based on the publication by Kupelian et al. [4]. This adjustment addressed the absence of the 50% probability within the upper bound of BCR estimates considered in the DSA. A probabilistic sensitivity analysis (PrSA) was not undertaken because several key parameters, particularly those informed by expert opinion and adapted literature estimates, lack the distributional data required to appropriately parameterize a PrSA. Conducting a PrSA using arbitrary or non-evidence-based distributions would risk introducing spurious precision and producing potentially misleading results. Instead, we conducted deterministic one-way sensitivity and scenario analyses across wide, conservative ranges to robustly test uncertainty. Given the model structure and available evidence, this approach provides a reliable assessment of parameter influence without overstating the certainty of the underlying data.

## 3. Results

### 3.1. Base Case Results

**Figure 2:**
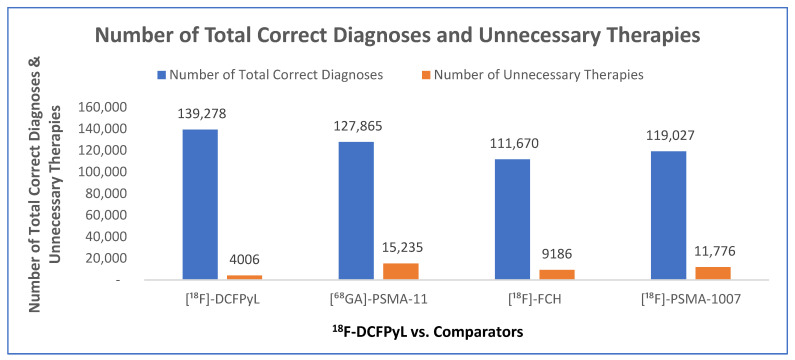
Number of total correct diagnoses and unnecessary therapies in BCR for [^68^Ga]-PSMA-11, [^18^F]-FCH, and [^18^F]-PSMA-1007 compared with [^18^F]-DCFPyL.

The CCA evaluated the economic and clinical impact of [^18^F]-DCFPyL in comparison with [^68^Ga]-PSMA-11, [^18^F]-FCH, and [^18^F]-PSMA-1007 PET/CT for detecting the BCR of PCa over a 5-year time horizon in the base case. This duration was selected to balance the relevance of the cost and treatment outcomes for practical decision-making while minimizing variability associated with longer timeframes. Additionally, the model allowed flexibility to adjust the time horizon between 1 and 10 years. The analysis demonstrated that [^18^F]-DCFPyL provided superior diagnostic accuracy across all comparisons, with 11,414 additional correct diagnoses (9% increase) relative to [^68^Ga]-PSMA-11, 27,609 additional correct diagnoses (25% increase) compared with [^18^F]-FCH, and 20,252 additional correct diagnoses (17% increase) over [^18^F]-PSMA-1007 (Figure 2).

[^18^F]-DCFPyL lowered the number of unnecessary therapies by 11,229, a reduction of 74%, compared with [^68^Ga]-PSMA-11. Similar reductions were observed when [^18^F]-DCFPyL was compared with [^18^F]-FCH and [^18^F]-PSMA-1007, with there being 5180 (56%) and 7771 (66%) fewer unnecessary therapies, respectively (Figure 2).

**Figure 3:**
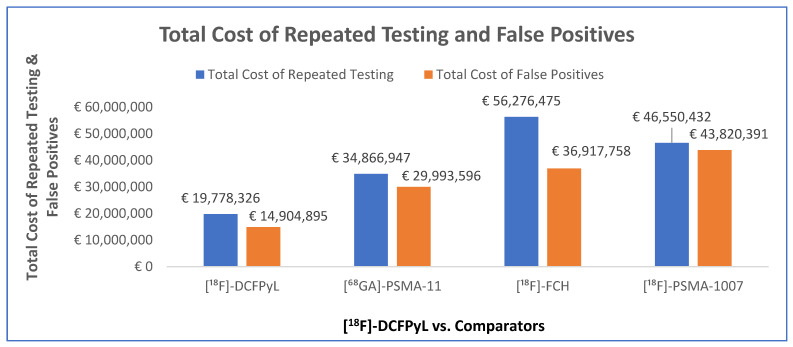
Total cost of repeated testing and false positives in BCR for using [^68^Ga]-PSMA-11, [^18^F]-FCH, and [^18^F]-PSMA-1007 compared with [^18^F]-DCFPyL.

Additionally, the model revealed significant cost savings with [^18^F]-DCFPyL in terms of repeated testing and false-positive diagnoses (Figure 3). For repeated testing, [^18^F]-DCFPyL resulted in savings of EUR 15,088,620 (43%) compared with 68Ga-PSMA200 11, EUR 36,498,149 (65%) compared with [^18^F]-FCH, and EUR 26,772,106 (58%) compared with [^18^F]-PSMA-1007. Similarly, the costs associated with false positives were markedly lower with [^18^F]-DCFPyL, yielding savings of EUR 15,088,701 (50%) compared to [^68^Ga]-PSMA-11, EUR 22,012,863 (60%) compared to [^18^F]-FCH, and EUR 28,915,496 (66%) compared to [^18^F] -PSMA-1007.

**Figure 4:**
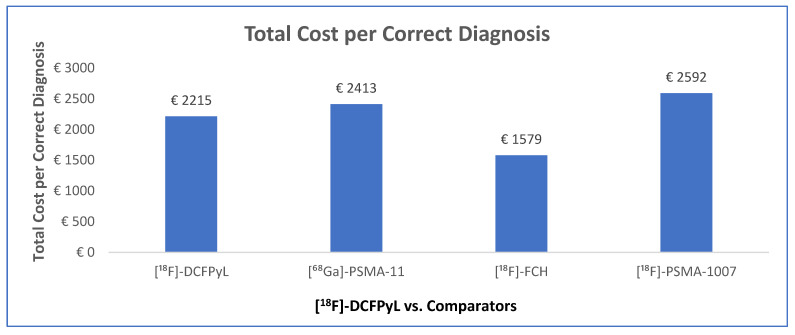
Cost–consequence (cost per correct diagnosis) in BCR for using [^68^Ga]-PSMA-11, [^18^F]-FCH, and [^18^F]-PSMA-1007 compared with ^18^F-DCFPyl.

The CCA indicated that the use of [^18^F]-DCFPyL compared with [^68^Ga]-PSMA-11 reduced the cost per correct diagnosis by EUR 198, an 8% decrease. When compared with [^18^F]-FCH, [^18^F]-DCFPyL led to an increase in the cost per correct diagnosis of EUR 635 (40%). Against [^18^F]-PSMA-1007, [^18^F]-DCFPyL achieved a reduction in cost per correct diagnosis of EUR 377 (15%) (Figure 4).

### 3.2. Sensitivity Analysis

For all the radiotracers considered, the DSA demonstrated that the total cost per correct diagnosis for [^18^F]-DCFPyL was highly sensitive to the key parameters, particularly the tracer costs and diagnostic accuracy. The cost of each radiotracer and their specificity and sensitivity had the greatest impact on the results (Supplementary Materials). Variations in the probability of BCR and the time horizon influenced the results, highlighting the critical role of diagnostic accuracy and tracer pricing in assessing the economic viability of [^18^F]-DCFPyL for detecting PCa recurrence. In the scenario analysis, across all radiotracers, the number of unnecessary treatments and the total cost of repeated testing nearly doubled when extending the time horizon from 5 to 10 years. On the other hand, a higher prevalence rate (50%) consistently led to a lower number of unnecessary treatments and a lower total cost of repeated testing across all radiotracers (Supplementary Materials). This trend underscored the cost implications of longer follow-up periods and higher disease prevalence.

## 4. Discussion

This decision analytic model-based analysis builds upon established economic evaluation frameworks for PET/CT imaging and extends prior work, such as the study of Jensen et al. [22], by incorporating a wider range of PET radiotracers for the detection of BCR relevant to current clinical practice in Spain. Test performance inputs for [^18^F]-DCFPyL were sourced from the OSPREY, CONDOR, and PYTHON studies, representing the most robust evidence available; however, the absence of direct head-to-head comparative trials and the heterogeneity of included studies regarding PSA thresholds, patient populations, and imaging protocols introduce uncertainty into the relative diagnostic performance estimates. Although the analysis synthesizes available data in a structured manner, future research using systematic meta-analysis or network meta-analysis, where feasible, would further strengthen comparative validity.

Within these evidence constraints, [^18^F]-DCFPyL demonstrated more favorable diagnostic characteristics compared with [^68^Ga]-PSMA-11, [^18^F]-FCH, and [^18^F]-PSMA-1007, which translated into fewer unnecessary therapies, reduced total costs associated with repeated testing, and lower costs related to false-positives. Additionally, the adoption of [^18^F]-DCFPyL could contribute to more effective and economically sustainable management strategies, making it a valuable option in evidence-based decision-making. These findings provide useful insight into short-term diagnostic and economic consequences, aligning with the scope of this work. However, there may be additional longer-term clinical and economic benefits that could not be evaluated due to the lack of robust longitudinal data, and future research incorporating endpoints such as survival or QALYs could build on this work as more evidence becomes available.

A probabilistic analysis was not conducted because the model is structured as a CCA rather than a full CEA with combined health outcomes. Many key inputs, particularly diagnostic accuracy estimates, were drawn from heterogeneous sources without consistent measures of variance suitable for probabilistic sampling. As a result, constructing meaningful joint probability distributions across all parameters was not feasible. Instead, extensive deterministic one-way sensitivity analysis was undertaken to test the robustness of results to parameter uncertainty. Future research using harmonized diagnostic accuracy data (e.g., through head-to-head trials or meta-analytic synthesis) would enable the development of appropriate probability distributions and support the inclusion of a PrSA. Incorporating QALYs and incremental cost-effectiveness ratios (ICERs) in terms of cost per QALY in such future models would also enhance comparability with conventional health technology assessment frameworks and increase policymaker utility.

Although the model was developed for the Spanish National Healthcare System, the approach may still be relevant to other countries with similar reimbursement mechanisms, radiotracer distribution systems, and PET/CT utilization patterns. That said, real-world adoption of [^18^F]-DCFPyL will depend on several system-level factors not captured in this analysis, including tracer availability, production and distribution logistics, access to PET/CT facilities, and reimbursement pathways. These practical considerations may influence uptake independently of the diagnostic and economic performance modeled.

Several model parameters, particularly cost components and treatment distributions, were informed by limited Spanish data, supplemented by non-Spanish sources when local evidence was lacking. While these inputs were tested in sensitivity analyses and found not to materially alter the study’s conclusions, reliance on external data may affect generalizability to the Spanish healthcare setting. Furthermore, only two Spanish clinical experts were consulted to inform model inputs and assumptions where empirical evidence was unavailable. Although these experts were selected for their experience in high-volume centers and provided detailed practice-based insights which was sufficient for the scope of this analysis, a larger panel could have offered additional perspectives. Nevertheless, sensitivity analyses indicated that varying these parameters within plausible ranges had limited impact on conclusions, suggesting that the findings remain directionally robust, but continued generation of Spanish-specific evidence would further strengthen the estimates.

Overall, this study represents the first attempt to compare all major PSMA radiotracers for BCR detection in PCa, using a unified modeling framework in Spain. While subject to the limitations inherent in heterogeneous data and the absence of head-to-head trials, the analysis provides a transparent and informative assessment of short-term diagnostic and economic implications of appropriate tracer selection. Future research integrating robust comparative clinical evidence through feasible meta-analytic synthesis, including QALYs, ICERs, and probabilistic analysis, would further strengthen decision-making support for policymakers and clinicians.

## 5. Conclusions

In summary, given the substantial costs of managing patients with PCa, healthcare decision-makers should evaluate PET imaging costs comprehensively, as accurate imaging is essential for guiding optimal care. This analysis suggests that [^18^F]-DCFPyL offers improved diagnostic accuracy, reducing the number of unnecessary therapies, resulting in cost savings related to repeated testing and false-positive diagnoses. Future cost-effectiveness studies incorporating QALYs would provide additional insights to further inform clinical decision-making.

## Figures and Tables

**Table 1:** Diagnostic performance of [^18^F]-DCFPyL and its comparators.

Author	Publication Year	Intervention	BCR Sensitivity Mean (Low; High)	BCR Specificity Mean (Low; High)
Pan et al. [28]	2021	[^18^F]-DCFPyL	0.91 (0.89; 0.93)	0.90 (0.87; 0.92)
Hope et al. [29]	2019	[^68^Ga]-PSMA-11	0.99 (0.96; 1.00)	0.76 (0.02; 1.00)
Marina et al. [30]	2015	[^18^F]-FCH	0.64 (0.51; 0.77)	0.76 (0.61; 0.91)
Liu et al. [31]	2022	[^18^F]-PSMA-1007	0.93 (0.89; 0.95)	0.71 (0.66; 0.75)

Abbreviations: BCR: biochemical recurrence.

## Data Availability

All data are incorporated in the article and its online Supplementary Materials (available online). The original contributions of this study are included in the article. Further inquiries can be directed to the corresponding author.

## Supplementary Materials

The following supporting information can be downloaded at: https://www.mdpi.com/article/10.3390/jmahp14010007/s1, Table S1: Proportion and Management of Localised, Regional and Metastatic Patients with Prostate Cancer; Table S2: Economic Inputs [32,33,34,35,36,37,38,39,40,41,42]; Figure S1: DSA results for cost-consequence (cost per correct diagnosis) for using (a) 68Ga-PSMA-11, (b) 18F-FCH, (c) 18F-PSMA-1007 compared with 18F-DCFPyl; Figure S2: Results for a scenario analysis by modifying the time horizon to 10 years, for (a) Number of futile treatments (b) Total cost of repeated testing.
