# Supplementary material for: Evaluating [18F]-DCFPyL for Detecting Prostate Cancer Recurrence: A Cost–Consequence Comparison with Alternative PET Radiotracers in Spain
Source: J Mark Access Health Policy. 2026 Jan 23;14(1):7. doi: 10.3390/jmahp14010007 (PMC12921982; doi:10.3390/jmahp14010007)
Supplement: Supplementary file 1 [file jmahp-14-00007-s001.zip › jmahp-3932932-supplementary/jmahp-3932932-supplementary.pdf]

Title

Evaluating ^{18}F-DCFPyL for Detecting Prostate Cancer Recurrence: A Cost-Consequence Comparison with Alternative Radiotracers in Spain.

Authors

Tiago Matos[1], Mrunmayee Godbole[2*], Rithvik Badinedi[3], Madhusubramanian Muthukumar[3], Marina Hodolic[4], Nicolas Tchouen[5], Anthony Berthon[6]

[1]Alira Health, Global Health Economics, Basel, Switzerland

[2]Alira Health, Global Health Economics, Barcelona, Spain

[3]Alira Health, Global Health Economics, London, United Kingdom

[4]Curium Pharma, Medical Affairs Europe, Paris, France

[5]Curium Pharma, Market Access, Paris, France

[6]Curium Pharma, Global Health Economics & Outcomes Research / Real-World Evidence, Paris, France

*Corresponding author

Email: godbolemrunmayee@gmail.com, mrunmayee.godbole@alirahealth.com

Table S1. Proportion and Management of Localised, Regional and Metastatic Patients with Prostate Cancer.

Treatments per Risk Group	Mean	Lower Bound	Upper Bound	Source
**Local High Risk**				
RT + ADT [Local (High risk)]	43%	34%	52%	Assumption validated by expert opinion
RP [Local (High risk)]	10%	8%	12%	
EBRT [Local (High risk)]	27%	22%	32%	
Brachytherapy [Local (High risk)]	20%	16%	24%	
**Regional**				
RT + ADT [Regional]	34%	27%	41%	Assumption validated by expert opinion
RP [Regional]	20%	16%	24%	
ADT [Regional]	26%	21%	31%	
Chemotherapy [Regional]	4%	3%	5%	
EBRT [Region]	17%	14%	20%	
**Metastatic Hormone Sensitive**				
Chemotherapy + NHA [mHSPC]	15%	12%	18%	Assumption validated by expert opinion
ADT + NHA [mHSPC]	85%	68%	100%	
**Metastatic Castration Resistant**				
NHA	52%	42%	62%	Assumption validated by expert opinion
Abiraterone [mCRPC]	54%	43%	65%	
Enzalutamide [mCRPC]	46%	37%	55%	
Chemotherapy [mCRPC]	36%	29%	43%	
Radium-223 [mCRPC]	4%	3%	5%	
Cabazitaxel [mCRPC]	5%	4%	6%	
Chemotherapy + NHA [mCRPC]	3%	2%	4%	

Abbreviations: RT: Radiotherapy; ADT: Androgen Deprivation Therapy; HIFU: High-Intensity Focused Ultrasound; RP: Radical Prostatectomy; EBRT: External Beam Radiotherapy; NHA: Novel Hormone Agent; PL: Pelvic Lymphadenectomy; mHSPC: Metastatic Hormone-Sensitive Prostate Cancer; mCRPC: Metastatic Castration-Resistant Prostate Cancer

Table S2. Economic Inputs.

Treatments/Diagnostics	Mean	Lower Bound	Upper Bound	Source
**Diagnostic Cost (unit cost)**				
^{18}F DCFPyl	€ 2,000	€ 1,800	€ 2,200	[32]
^{18}F-FCH	€ 1,143	€ 1,029	€ 1,258	[33]
^{68}Ga-PSMA-11	€ 2,000	€ 1,800	€ 2,200	[32]
^{18}F-PSMA-1007	€ 2,000	€ 1,800	€ 2,200	[34]
**Treatment Cost (unit cost)**				
Radiotherapy	€ 2,790	€ 2,232	€ 3,348	[35]
Brachytherapy	€ 10,090	€ 8,072	€ 12,108	[36]
External Beam Radiation Therapy	€ 4,467	€ 3,574	€ 5,360	[37]
Radical Prostatectomy	€ 7,200	€ 5,760	€ 8,640	[35]

Androgen Deprivation Therapy	€ 780	€ 624	€ 936	[35]
Chemotherapy	€ 1,291	€ 1,033	€ 1,549	[38]
Radium-223	€ 27,195	€ 21,756	€ 32,634	[39]
Cabazitaxel + Prednisone	€ 11,198	€ 8,958	€ 13,438	[40]
Abiraterone + Prednisone	€ 37,740	€ 30,192	€ 45,288	[41]
Enzalutamide	€ 35,224	€ 28,179	€ 42,269	[41]
**PCa Confirmation Cost**				
Immunohistochemistry	€ 1,322	€ 1,182	€ 1,462	[42]

Abbreviations: PCa: Prostate Cancer

Figure S1 *DSA results for cost-consequence (cost per correct diagnosis) for using (a) 68Ga-PSMA-11, (b)^{18}F-FCH, (c) ^{18}F-PSMA-1007 compared with ^{18}F-DCFPyl.*

(a)

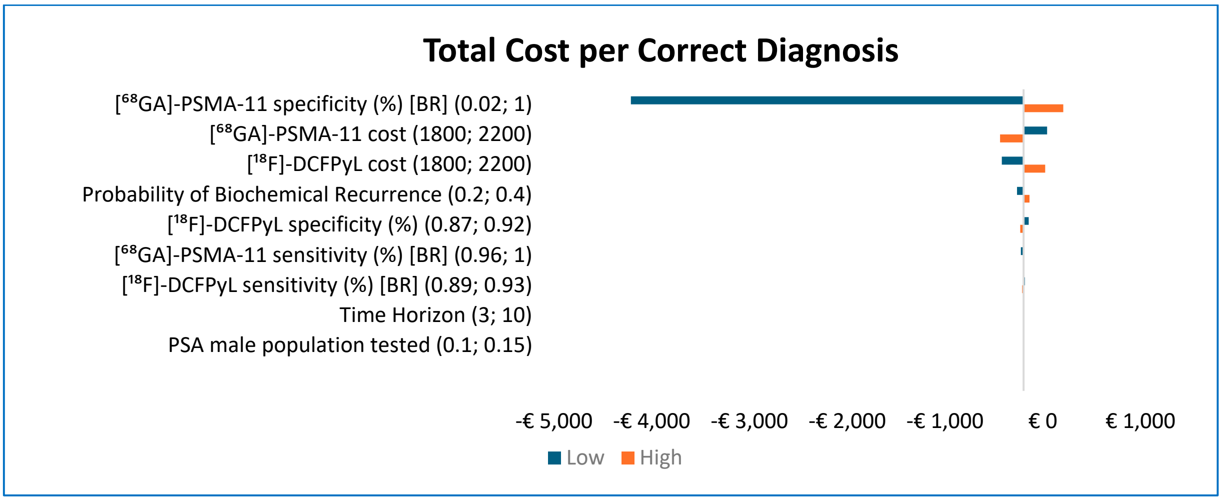

(b)

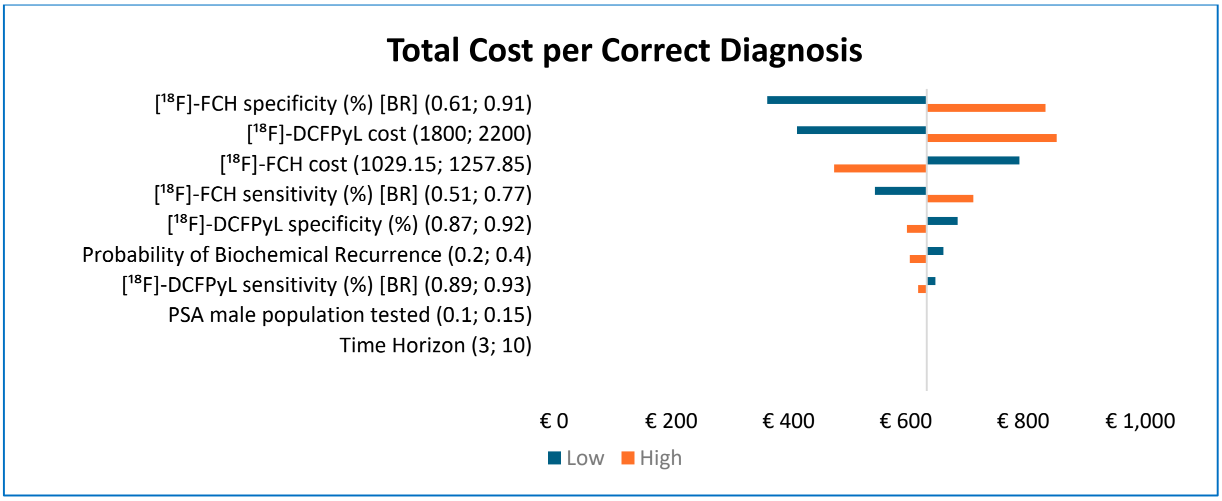
Total Cost per Correct Diagnosis
[18F]-FCH specificity (%) [BR] (0.61; 0.91)
[18F]-DCFPyL cost (1800; 2200)
[18F]-FCH cost (1029.15; 1257.85)
[18F]-FCH sensitivity (%) [BR] (0.51; 0.77)
[18F]-DCFPyL specificity (%) (0.87; 0.92)
Probability of Biochemical Recurrence (0.2; 0.4)
[18F]-DCFPyL sensitivity (%) [BR] (0.89; 0.93)
PSA male population tested (0.1; 0.15)
Time Horizon (3; 10)
€ 0
€ 200
€ 400
€ 600
€ 800
€ 1,000
Low
High

(c)

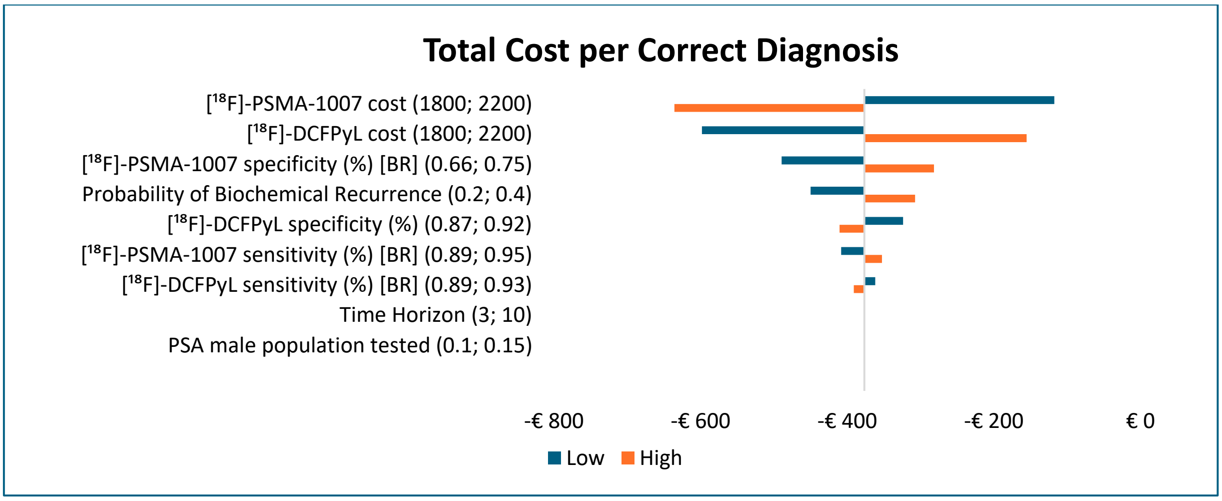

Figure S2 *Results for a scenario analysis by modifying the time horizon to 10 years, for (a) Number of futile treatments (b) Total cost of repeated testing.*

(a)

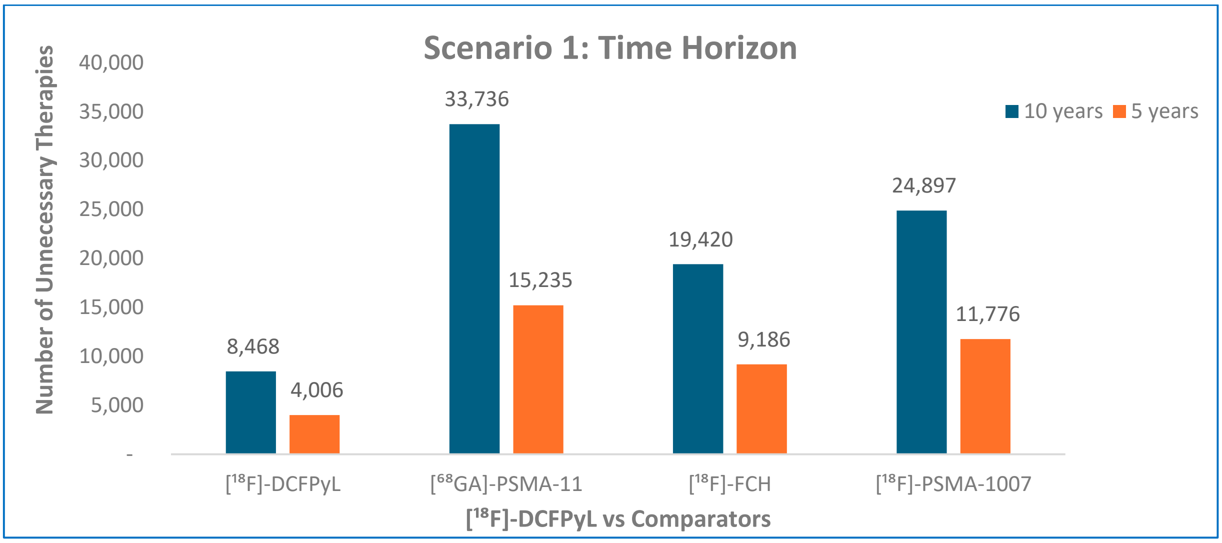

(b)

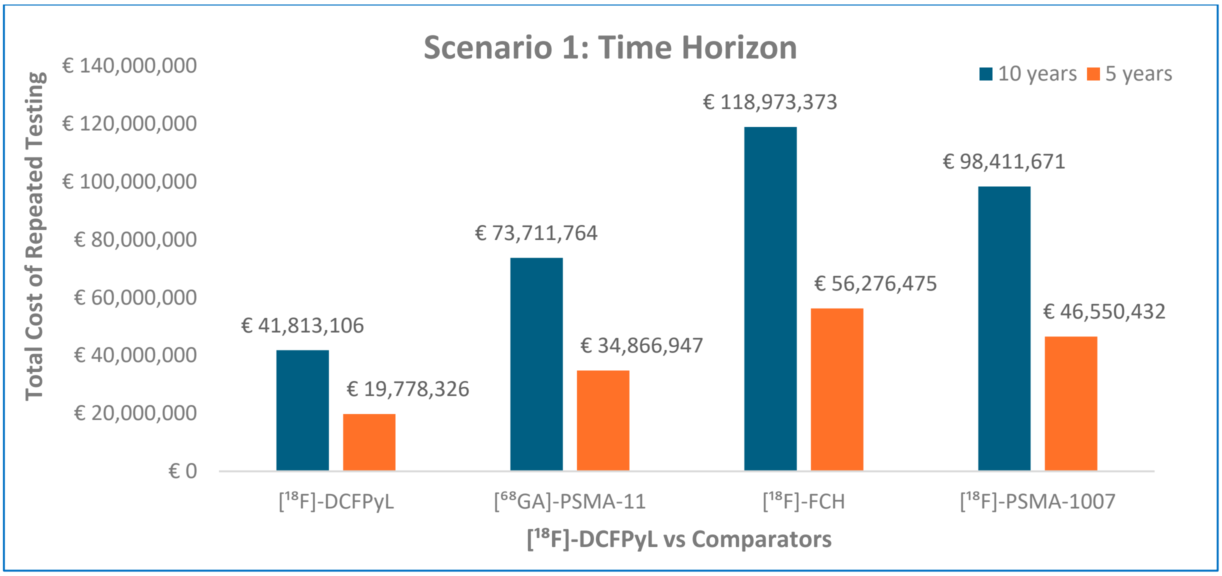

Figure S3 *Results for a scenario analysis by modifying the prevalence to 50%, for (a) Number of futile treatments (b) Total cost of repeated testing.*

(a)

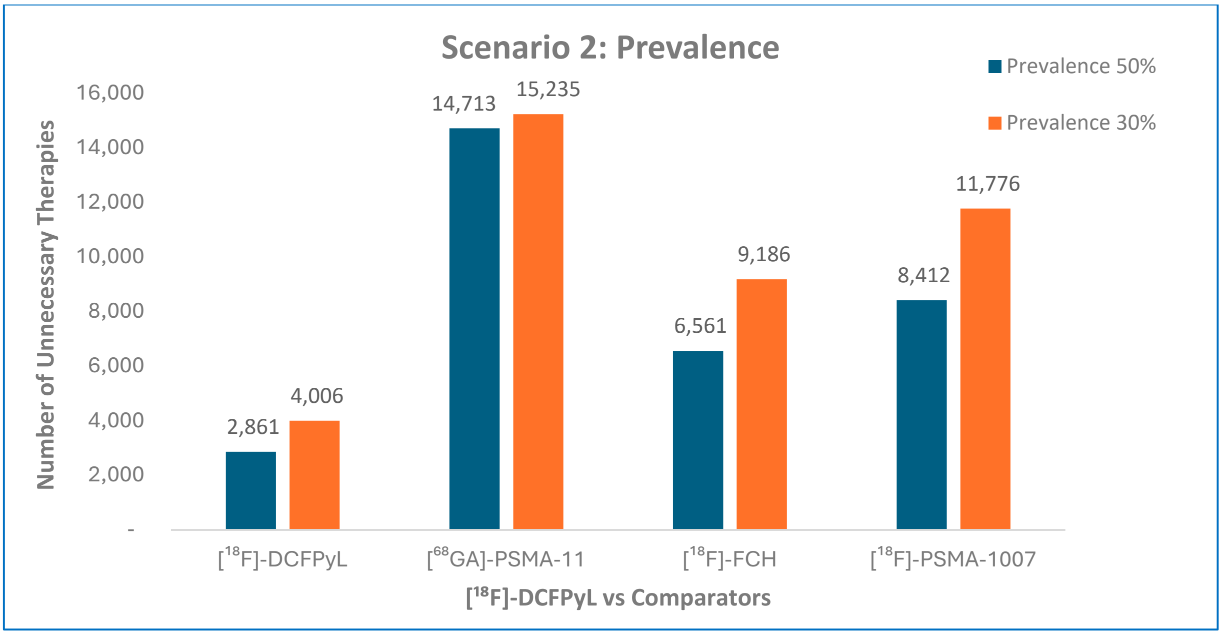

(b)

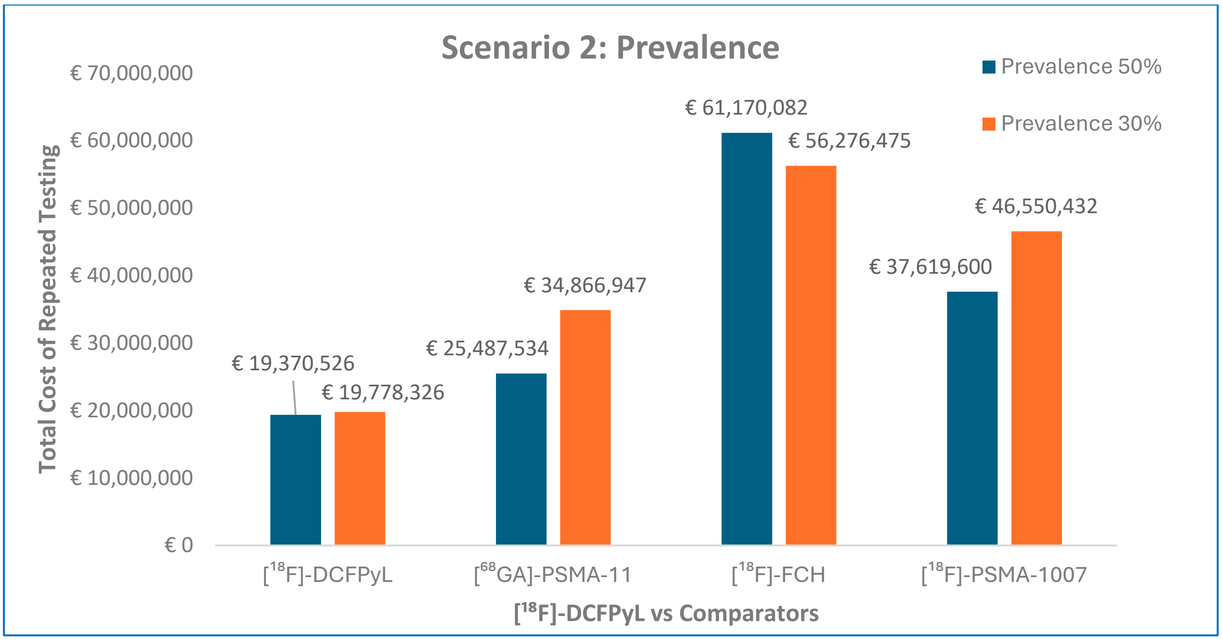
Scenario 2: Prevalence
Prevalence 50%
Prevalence 30%
Total Cost of Repeated Testing
€ 70,000,000
€ 60,000,000
€ 50,000,000
€ 40,000,000
€ 30,000,000
€ 20,000,000
€ 10,000,000
€ 0
€ 19,370,526
€ 19,778,326
€ 25,487,534
€ 34,866,947
€ 61,170,082
€ 56,276,475
€ 37,619,600
€ 46,550,432
[18F]-DCFPyL
[68GA]-PSMA-11
[18F]-FCH
[18F]-PSMA-1007
[18F]-DCFPyL vs Comparators
